# An estimate of the monthly cost of two major mental disorders in an Indian metropolis

**DOI:** 10.4103/0019-5545.31576

**Published:** 2006

**Authors:** P. Sharma, S.K. Das, S.N. Deshpande

**Affiliations:** * Clinical Psychologist, Department of Clinical Psychology, Institute of Human Behaviour and Allied Sciences, Dilshad Garden, Delhi 110095; ** Psychiatric Social Worker, Department of Psychiatry, Dr Ram Manohar Lohia Hospital, Park Street, New Delhi 11000; *** Senior Psychiatrist and Head, Department of Psychiatry, Dr Ram Manohar Lohia Hospital, Park Street, New Delhi 11000

**Keywords:** Economic costs, families, care-giving, schizophrenia, bipolar disorder

## Abstract

**Background:**

The components of high cost of treating a chronic psychiatric illness are: long-term continuous treatment consisting of consultation and medication costs, traveling to the treatment centre and taking time off from work for both patient and caregiver. Apart from direct treatment costs, expenditure of time in care-giving results in indirect costs. All these costs are borne by families as the sufferer may be unable to work.

**Aim:**

To estimate the cost of treatment of chronically ill patients at home, in terms of the above parameters.

**Methods:**

The sample consisted of 117 subjects of either sex in the age range of *18* to *60* years, ill for at least one year, diagnosed as schizophrenia (*n*=95) or bipolar disorder (*n*=22, a comparison group) who agreed to participate in the study along with at least one caregiver. The tools used were the Diagnostic Interview of Genetic Studies and Economic Burden Questionnaire administered to both the subject and the caregiver.

**Results and conclusions:**

The costs of treatment were found to be high but with wide variations in the range. Costs for bipolar disorder were somewhat higher than those for schizophrenia at least for the period of study. Demographic differences between subjects and caregivers were present.

## INTRODUCTION

The family has a significant role in the treatment and prognosis of mental illness of an individual. Initially the role was viewed as contributory in the aetiology of illness, whereas the focus of current research has shifted towards the understanding of the effect of mental illness on the family. Hoenig and Hamilton^1^ pioneered the concept of objective and subjective burden experienced by family members of the mentally ill person and tried to differentiate between the two. The presence of a mentally ill person at home affects all family members, problems occur in the areas of health, recreational activities, social and marital relations, and above all finances. Families of psychiatric patients thus experience substantial burden which indicates the breakdown of reciprocal arrangements that people maintain in their relationships, such that one person is doing ‘more than their fair share’.^2^ A large majority of persons with mental illness either remain unemployed or underemployed which further adds to the financial burden of the families. In two different studies it was found that the financial burden experienced by the families were up to the extent of difficulty in visiting hospitals (58%,^3^ 55%^4^).

In the 1960s, the shift from hospital- to community-based care was supposed to reduce the load on hospitals, help early recovery and prevent chronic handicap among mentally ill persons as well as effect substantial savings for indoor hospital care.^5^ Many countries launched large-scale community mental health programmes without assessing the burden that the family had to face and possible effect on other family members.^6^ So far only a few attempts have been made to assess the type and degree of burden placed on families of sufferers treated at home in India.

Although research on actual cost in treating widely prevalent diseases has appeared in medical literature,^7^–^11^ less work has been carried out in India on economic burden of mental illness.^3^,^12^ While terms such as direct costs, indirect costs (quantified in India to some extent^3^) and soft costs^7^ are used when healthcare economics is discussed, actual costs of mental illness have not been studied in detail. Direct costs involve cost of travelling, taking time off from work for both the patient (if employed) and caregiver, and cost of psychotropic drugs (long duration of treatment increases cost). Generic drugs may or may not cost less than the branded ones.^13^–^15^

The present study focused on the cost of chronic major mental illnesses to family and patient. One problem in carrying out such studies is difficulty in recollecting approximate expenses incurred on various treatment modalities, travel, cost of drugs, wages lost and other expenses from the very onset of the illness. To circumvent this problem, the present study focused on reviewing details of treatment expenditures for the past 3 months only. Expenditure included expenses related to treatment by traditional healers, private practitioners, outpatient treatment, drugs taken with frequency and duration, loans taken and also time spent by the two main caregivers consequent to patient's illness.

## METHODS

The study was conducted between November 2002 and March 2003 in the Department of Psychiatry, Dr Ram Manohar Lohia Hospital (RMLH), New Delhi, a tertiary care general hospital that provides both inpatient and outpatient care free of consultation cost. Those eligible for inclusion were subjects clinically diagnosed as having schizophrenia (Sz) or bipolar affective disorder (BAD),^16^ within ages 18 to 60 years with duration of illness of more than 1 year. Those with suspected organic causes or drug abuse and those below average intelligence were excluded. All parameters for obtaining measures of economic burden were calculated for the past 3 months from the date of interview. Reference costs for drugs were taken from the *Drug Index.*^17^

The project was approved by the Ethics Committee of RMLH. All psychiatrists working in the Department of Psychiatry, RMLH first informed eligible subjects and caregivers about the study. It was a purposive sample as only subjects who fulfilled the inclusion criteria and agreed to participate in the study were included after a written informed consent by both subjects and their caregivers. Almost all the subjects had been receiving treatment from the hospital for 6 months or more. A total of 117 subjects along with their caregivers were included for the final analysis, out of which 95 were diagnosed as having Sz and 22 as having BAD.

### Tools

1. *Diagnostic Interview of Genetic Studies (DIGS)* Section A (Demographics) and Section E (Overview of Psychiatric Disturbance) only, were used. Developed by Nurnburger^18^ the instrument has been translated into Hindi.^19^
2. *Economic Burden Questionnaire* based on the Client Social Demographic and Service Receipt Inventory^11^ modified by Murthy (personal communication 2000), was administered to assess the economic burden on the subject and caregiver. The original semi-structured questionnaire used in India^20^ to calculate economic cost of various services and cost of informal care-giving helps to compute direct, indirect and intangible costs of mental health care. There were 16 questions relating to utilization of various services during the previous 3 months including number, cost and time required for consultations, consultation with primary healthcare providers, traditional healers and hospital services, medication used, and support and help from family and friends.

### Measures of Cost

Economic analysis was carried out from the point of view of both subjects and caregivers. The data were analysed for money and time cost, as follows.

#### Direct treatment cost (money) for preceding 3 months

1. *Consultation cost:* Expenditure on travel to and from consultations, fees paid per visit, number of visits by subject and caregiver together or separately were calculated to assess money spent on consultation on an average visit during past 3 months.
2. *Medication cost:* Money spent on medication during the past 3 months was calculated. From the prescriptions available with the patient/caregiver, a drug list was drawn, that included all medications prescribed for the past 3 months along with their potency, dosage and duration. Cost per tablet was calculated with the help of Indian *Drug Index.*^17^ The local pharmacist was contacted for prices of tablets not mentioned in the *Drug Index.* Where generic names were prescribed the price of the cheapest brand was used for calculation. All prices were taken at the rate of maximum retail price mentioned on the tablet foil. Some drugs could have also been sold with local taxes added—an extra cost, which was not added to the total cost. Hence the cost mentioned here could be less than the actual cost borne by subjects. At the end, the average number of medications being taken by a patient and an average cost was calculated from the above list.
3. *Raising extra money:* Any loans and /or goods sold by the subject or the caregiver for the purpose of treatment during the past 3 months were also noted. While a few caregivers had sold goods or taken loans, since it was not exclusively for treatment, these amounts were NOT taken into consideration.

### Indirect treatment costs (time and money)

This included time spent on visits to different services by the subject and the caregiver who accompanied the subject. It also included extra hours spent by the two main caregivers for subject's care or taking on subject's responsibilities.

1. *Time for consultation:* Average time spent by the subject in travelling, waiting and meeting the healer/doctor for various services was calculated. Since most cases were accompanied by at least one caregiver, time spent by caregiver was also added.
2. *Time for care-giving:* Number of hours spent per week by 2 main caregivers for subject's care or in carrying out subject's responsibilities was noted. Caregiver 1 spent maximum time looking after the subject; Caregiver 2 spent less time than Caregiver 1 but more than other family members. Although the questionnaire classified care-giving in 4 different areas (child care, help around the house, help outside the house and other help), for simplicity of calculation all 4 areas were added together even though some responsibilities might have been carried out simultaneously (e.g. child care and household work).
3. *Income loss in informal care-giving:* The family of a mentally ill person has to not only look after the subject but also do his/her share of work (informal care-giving). To calculate the cost (in Rupees) of total time spent in informal care-giving for caregivers who were housewives, students or unemployed with no clear income, notional income was taken as Rs 100 per day.^20^ Hourly income was calculated keeping average work time of 8 hours per day, 6 days a week. Total time spent in care-giving in hours per month was then multiplied by hourly income to obtain monthly income loss.
4. *Job loss due to care-giving:* If a caregiver had to give up his or her job to look after the subject, monetary loss was also added to the economic burden. However, in our sample no caregiver needed to give up his/her job to look after the subject.

## RESULTS

The final sample consisted of subjects suffering from schizophrenia (Sz 81%) and a smaller group suffering from bipolar affective disorder (BAD 19%). The majority of caregivers were females, older than the subjects (80% of subjects were <45 years, 55% of caregivers were >45 years), married, and less educated than subjects. While 29% of caregivers were unemployed, as compared to 49% of subjects, half of the caregivers were parents (50.4%), 36% were spouses. Most of the subjects lived with their families: 51% either with their parents or children, 38% with spouses, 11% sharing home with other relatives, siblings or others. None stayed alone. While 71% of the caregivers had no health problems, 23% suffered from one medical illness and 6% from two or more. Most families were from lower middle class; 49% had a monthly income below Rs 5000 and 28% earned between Rs 5000 and Rs 10,000 per month. Most of the families lived in urban areas (77%), 16.5% in suburban and 6% in rural areas. Further demographic details of the sample of patients as well as caregivers are available with authors and have been presented elsewhere.^21^,^22^

In terms of travel costs and fees for consultation, outpatient services were the cheapest. No consultation fees needed to be paid at RMLH, average amounts spent on travel (Sz Rs 66 per person, BAD Rs 97 per person) were also relatively low, depending on where the patient travelled from (Table 1). For availing primary care, a lesser amount of Rs 56 as fees, and less than Rs 8 as travel were spent by Sz subjects and families. Fees paid to the primary care facility were Rs 129 and travel cost Rs 215 per person/visit for BAD subjects. Sz subjects and their caregivers spent Rs 467.78 in preceding 3 months on fees for the traditional healer and another Rs 481.67 on travelling for their visits; BAD subjects/families who had gone to traditional healers had spent Rs 2832 for fees and another Rs 1675 on travelling per person/visit.

**Table 1:** Average monthly expenditure for travelling and consultation (in Rs)

	Average total fee	Average total travel expenditure per person
**Schizophrenia (Sz)**		
91 subjects + 74 caregivers (Outpatient service at RMLH)	0	65.92±110.17
10 subjects + 10 caregivers (primary care facility)	56.0±21.95	7.50±18.74
6 subjects + 6 caregivers (traditional healer)	467.78±998.31	481.67±991.69
**Bipolar affective disorder (BAD)**		
22 subjects + 16 caregivers (Outpatient service at RMLH)	0	97.35±200.28
2 subjects + 2 caregivers (primary care facility)	129.16±5.89	215.00±285.19
3 subjects + 3 caregivers (traditional healer)	2832.77±4764.33	1675.55±2879.06

The expenses on travel and time spent in travelling, waiting and meeting the service providers were doubled in all cases where caregiver accompanied the subject.

Sz subjects were taking approximately 4 (3.71) types of medications per month, ranging from 1 to 14 medicines, i.e. some subjects had been on a single type of medication only whereas others were tried on many, as many as 14 in past 3 months in some cases (Table 2). The average number of medications in BAD subjects was also 4; however, some of them did not take any medication at all in the past 3 months. The range was from 0 to 15 drugs in the past 3 months for BAD. Though both types of subjects were on an average taking 4 types of medications per month, BAD subjects spent more on the drugs (Sz average monthly cost Rs 288, and BAD Rs 364).

**Table 2:** Average amount spent by subjects on medicines in one month (in Rs)

Diagnosis	Average no. of prescribed drugs (Mean±SD)	Average cost of drugs per month* (Mean±SD, in Rs)
Schizophrenia (Sz)	3.71±2.38	288.12±231.69
Bipolar affective disorder (BAD)	4.23±3.23	364.33±294.08

* Exclusive of local taxes

In all cases, both the subjects and the caregivers were asked about the time spent on consultation. The estimates were almost the same hence the value given by the caregiver was taken for calculation (Table 3). The expenses on travel and time spent in travelling, waiting and meeting the service provider were doubled in all cases where a caregiver accompanied the subject. All expenses were much higher for BAD families.

**Table 3:** Time (in hours) spent in availing different treatment services per month

Services used	Average travel time (hours) /person/trip*	Average wait time (hours) /person/trip	Average number of consultations/month	Average meet time (hours)
**Schizophrenia (Sz)**				
Outpatient service S=91, C=74	1.98±1.46	1.89±3.00	1.18±1.00	0.20±0.15
Primary care facility** S=10, C=10	0.77±1.08	0.43±0.75	1.57±2.99	0.20±0.26
Traditional healer S=6, C=6	10.05±19.60	1.34±2.05	2.28± 1.88	2.85±4.56
**Bipolar affective disorder (BAD)**			
Outpatient service S=22, C=16	4.86±11.79	2.53±2.15	2.21±3.91	0.42±1.00
Primary care facility** S=2, C=2	2.25±2.71	1.54±0.76	4.83±4.94	1.70±0.53
Traditional healer S=3, C=3	45.46±76.11	12.44±18.20	6.00±9.24	1.57±2.25

S: subject, C: caregiver

* Cost doubled when caregiver accompanied subject

** Primary care facility included non-psychiatric medical practitioners

For consulting a specialist, after travelling for nearly 2 hours, Sz subjects and their caregivers had to wait for another 2 hours, or go through various administrative formalities such as registration, before they could meet the doctor. In case of BAD subjects the travel time was doubled (4 hours) but waiting time was almost the same. Sz subjects met the psychiatrist for about 11 minutes, and BAD subjects for about 21 minutes. Sz subjects (91), often accompanied by their caregivers (74), made at least one visit to the psychiatrist in the preceding month. BAD subjects needed to make at least 2 visits and had to spend much more time waiting in the outpatient services for consultations (of 22 BAD subjects, 16 were accompanied by a relative). All time estimates were based on figures given by subjects and caregivers.

Some patients needed to consult primary care physicians for various reasons. Travel time was relatively less for consulting primary care (i.e. non-psychiatric physicians); for Sz subjects it was ¾ of an hour, and for BAD subjects 2¼ hour, waiting time being ½ and 1½ hours, respectively. Relatively more time was spent by the subject and the caregiver waiting for the doctor in outpatient services (Sz 1.89 hours and BAD 2.3 hours) in comparison to primary care (Sz 0.4 hours, BAD 1.54 hours). However, only a fraction of subjects availed of primary care: 10 subjects and caregivers each for Sz (2 visits), and 2 subjects and caregivers each for BAD (5 visits). Primary care physicians spent as much as 1.5 hours with BAD subjects, and only one-fifth of an hour with Sz subjects.

The few patients (Sz 6/91 making 2 visits, BAD 3/22 making 6 visits) who wanted to consult a traditional healer had to travel longer distances and wait longer for consultation to travel (10 hours and 46 hours) and to wait (1.3 hours and 12.4 hours, respectively) for Sz and BAD subjects—perhaps because ‘specialist healers’ are now scarce within boundaries of Delhi. For Sz this was almost five times the duration they spent to visit outpatient services (2 vs. 10 hours) and by BAD families 8 times (6 vs. 46 hours) during one month. Traditional healers satisfied their customers however by spending much more time with them (Sz and BAD—2.9 and 1.6 hours, respectively). Nevertheless, only 6 of 91 (5.5%) Sz families and 3 of 22 (13.6%) BAD families actually visited traditional healers during the preceding month.

While subjects were always accompanied by caregiver/s for visits to the traditional healer, many visited the psychiatrist and primary care physician alone (17 of 95 Sz subjects, 5 of 22 BAD subjects), thus reducing the costs. Overlap in cases seeking more than one service was present.

Average time spent by caregivers taking care of the subject and his/her duties and subsequent income loss (Table 4) was also substantial. While the majority (46 of 86 for Sz and 13 of 21 for BAD) caregivers were housewives and care-giving was an extra duty, employed caregivers also had to spend extra time at home. For Sz subjects, caregivers spent an average minimum of 33 hours and a maximum of 124 hours, losing between Rs 567 to Rs 2000 per month. For BAD subjects, the corresponding figures are 50–153 hours, and loss of income of Rs 600–2750. The cost of this informal care-giving further increases the cost of mental illness.

**Table 4:** Hours spent by first caregiver and income loss in one month by first caregiver

Caregiver's occupation	Total hours spent in one month	Total income loss in one month (Rs)	Caregiver's occupation	Total hours spent in one month	Total income loss in one month (Rs)

Sz (*n*=86)	Mean±SD	Mean±SD	Bad (*n*=21)	Mean±SD	Mean±SD
Housewife (46)	95.65±76.99	1195.65±962.45	Housewife (13)	152.62±96.37	1907.69±1204.65
Service (18)	109.11±88.14	1990.09±1831.38	Employed (2)	50.00±53.74	600.00±70.710
Pensioner (10)	76.4±61.49	1426.27±1286.08	Pensioners (3)	68.00±24.98	1894.33±1121.36
Student (3)	54.67±6.11	683.33±76.37	Unemployed (2)	84.00±45.25	1050.00±565.68
Unemployed (3)	124±54.11	1550±676.38	Business (1)	220.00	2750.00
Business (3)	33.33±14.04	566.66±284.8
Other (3)	96.1±96.99	1300±1217

## DISCUSSION

Since the sample for BAD patients was small, no attempt was made to compare costs between the two samples. Most families in the present sample earned less than Rs 5000 per month and depended mainly on free government facilities for treatment. A large majority also belonged to the urban area, where the cost of living is itself very high. Some of the caregivers suffered from one or more medical illnesses leading to increased visits to the physician.^23^ While no cost was incurred on outpatient consultation fees, travel costs were quite high and doubled because caregiver was also expected to accompany the subject.

Subjects and caregivers spent much more time travelling and waiting than in actually meeting the psychiatrist in the hospital as the numbers of patients in government hospitals were far higher than the available specialist manpower. This emphasizes the need to have more mental health centres in the community so that the families do not have to travel long distances to seek treatment and this in turn would reduce to some extent the existing economic burden of care on families.

The visits made by persons with schizophrenia were fewer perhaps as most of them were stable, requiring less frequent visits unlike the BAD subjects who made at least two visits to the hospital per month. This finding is not to be generalized as in the present paper no attempt was made to compare the two groups. A couple of BAD subjects were from outside Delhi which led to a significantly higher travelling cost. During acute exacerbations, BAD subjects might have been more disruptive, needing to travel by private vehicles accompanied by several caregivers. A few families availed of more than one treatment service such as visiting primary care physicians or travelling long distances to traditional healers.

Though the amount spent by the families on medicines in past 3 months was not found to be very high but these costs could not be viewed in isolation. Other expenditure such as cost of travel, and the time and money spent in seeking other services also added to the overall burden. Prices of medicines have also shot up in the past two years. Cost mentioned was only in respect of past 3 months in 2002–2003. With frequent increase in costs and considering expenditure across the lifespan, actual costs burgeon over the years. Experience in other countries indicates that Sz is an expensive disorder right across the adult lifespan, and expenditure increases with age.^24^ Research has also shown that frequent relapses increase the cost of care in both BAD as well as Sz up to four-fold.^25^ Hence, one of the chief goals of treatment should be to prevent relapse by using more effective medicines or increasing their dose or numbers. Patel and Andrade^20^ suggested that it would be highly beneficial to keep the overall drug costs low and patent laws modified as all the other three strategies mentioned were most likely to increase the expenditure.

BAD caregivers spent more time in informal care-giving possibly because BAD sufferers were more disruptive (during mania) or caused more worry (during depression) to their caregivers. These sufferers were also therefore possibly given more time by the consultants as their illness demanded more time. On an average, an expenditure of 4 hours in consultation, and from 33 to 124 hours in care-giving was expended for care of persons with Sz or for taking care of their duties. For BAD the corresponding figures were 16 hours in consultation and 50-153 hours in care-giving. Costs incurred for medicines and consultations for Sz were Rs 353 (consultation and medication) and for BAD Rs 558. Care-giving costs varied from Rs 500 to Rs 2000 depending on the earning capacity of the caregiver.

A few subjects visited other primary medical facilities; these visits were more expensive in terms of time rather than money. Much larger amounts were spent on visits to traditional healers, indicating the desperation for disease cure or control. Waiting time as outpatients was much higher than consultation time; and this might have increased frustration and turned the family towards traditional healers who spent much more time with the patient.

For BAD and schizophrenic subjects, key caregivers were housewives, unemployed persons at home, pensioners and service goers who spent a lot of time in looking after the subject. The maximum monetary loss faced by these primary caregivers was computed to be Rs 1500–2000 per month. Caregivers also lost money due to time taken off work for visiting treatment facilities which could not be quantified.

## CONCLUSION

It is not only direct but also indirect costs that play a significant role in increasing economic burden on caregivers. Since travel contributed the largest share of cost both in terms of time and money, having treatment facilities distributed in the community, fewer consultations during the stable part of illness and training the primary care physician to manage stable illness may reduce costs and indirectly the burden. Patent regimes need to vigilantly bring down costs. Caregiver burden may also reduce with ancillary respite care, day care, sheltered work or part-time hospitalization. This reduces the time spent in informal care-giving. It would help the affected person to get rehabilitated in society. Sz subjects themselves (63%) expressed the desire for getting some employment (information available with authors). Finally, effective treatment and relapse prevention should be the primary aim as this will reduce costs and burden all around. Some other suggestions include longer medication supplies be given to subjects in government facilities; to those on maintenance treatment or are stable to avoid frequent visits and expenditure. Increasing awareness about mental illness and its treatment would not only reduce the stigma but also save the caregivers from spending heavily on the traditional/faith healers in terms of their time and money. It would also facilitate early initiation of treatment.
